# Contrasting physiological and pathological tau phosphorylation

**DOI:** 10.1002/alz70855_107479

**Published:** 2025-12-25

**Authors:** Nima N Naseri, Taylor L Tomlinson, Marie Shimogawa, E. James Petersson, Ophir Shalem, Elizabeth Rhoades

**Affiliations:** ^1^ University of Pennsylvania, Philadelphia, PA, USA; ^2^ Children's Hospital of Philadelphia at University of Pennsylvania, Philadelphia, PA, USA

## Abstract

**Background:**

Although phosphorylation to the microtubule‐associated protein tau is strongly correlated with its aggregation and neurodegeneration in Alzheimer's disease, tau is also abundantly phosphorylated during normal, physiological development. Yet, tau phosphorylation is not associated with toxicity in developing brains. This divergence in the effects of phosphorylation to tau at different ages makes it of interest for further study.

**Method:**

Here, we compare site‐specific effects of tau phosphorylation in developing and aged mouse brains. We further characterize combinatorial combinations of phosphorylation to tau in embryonic neurons to identify specific tau phospho‐proteoforms. Lastly, we investigate the site‐specific regulation of tau phosphorylation under physiological conditions. These experiments were achieved using mouse aging studies, sub‐cellular fractionations assays in human iPSC‐derived neurons and cell‐free biophysical assays using semi‐synthesized phosphorylated tau.

**Result:**

Although phosphorylation to tau is associated with insoluble tau in aged brains, we found that nearly 100% of phospho‐tau is soluble in neonatal mouse brains. Furthermore, we identified the cellular‐mechanism via which specific sites in tau are phosphorylated under physiological conditions of neuronal axon growth and collapse. Lastly, we identified specific combinatorial patterns of phosphorylation that are unique to the physiological state and thus distinct from disease‐specific tau proteoforms.

**Conclusion:**

Our findings highlight the possibility of differential regulation of tau phosphorylation in the developing and adult brains. Overall, by contrasting physiological and pathological phosphorylation patterns to tau, we discern tau proteoforms that are unique to the disease state, and thus strong candidates for therapeutic clearance.

